# Supplementary material for: Allosteric Inhibitors of Zika Virus NS2B-NS3 Protease Targeting Protease in “Super-Open” Conformation
Source: Viruses. 2023 Apr 30;15(5):1106. doi: 10.3390/v15051106 (PMC10224173; doi:10.3390/v15051106)
Supplement: Supplementary file 1 [file viruses-15-01106-s001.zip › viruses-2266135-supplementary.pdf]

Allosteric Inhibitors of Zika Virus NS2B-NS3 Protease Targeting Protease in "Super-Open" Conformation

Ittipat Meewan [1,2,†], Sergey A. Shiryaev [3,†], Julius Kattoula [2], Chun-Teng Huang [3], Vivian Lin [3], Chiao-Han Chuang [3], Alexey V. Terskikh [3,*] and Ruben Abagyan [2,*]

1 Institute of Molecular Biosciences, Mahidol University, Nakhon Pathom 73170, Thailand
2 Skaggs School of Pharmacy and Pharmaceutical Sciences, University of California San Diego, La Jolla, CA 92093, USA
3 Sanford-Burnham-Prebys Medical Discovery Institute, La Jolla, CA 92037, USA
* Correspondence: terskikh@sbpdiscovery.org (A.V.T.); rabagyan@health.ucsd.edu (R.A.)
† These authors equally contributed to this work.

SUPPLEMENTARY DATA

Table S1. Code names, structures, and ICM binding scores of initial ten candidates from virtual screening against ZIKV NS2B-NS3 protease.

Code	Structure	Chemical formula	Name	ICM Docking Score
IRA01	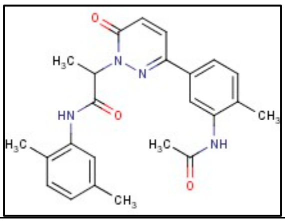	$C_{24} H_{26} N_4 O_3$	2-[3-[3-(acetylamino)-4-methylphenyl]-6-oxo-1(6H)-pyridazinyl]-N-(2,5-dimethylphenyl)propanamide	-35.69
IRA02	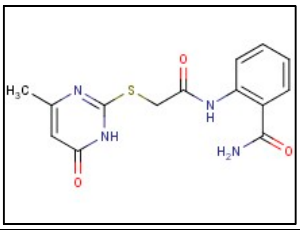	$C_{14} H_{14} N_4 O_3 S$	2-({[(4-methyl-6-oxo-1,6-dihydro-2-pyrimidinyl)thio]acetyl}amino)benzamide	-34.07
IRA03	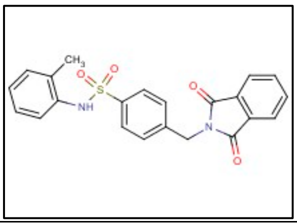	$C_{22} H_{18} N_2 O_4 S$	4-[(1,3-dioxo-1,3-dihydro-2H-isoindol-2-yl)methyl]-N-(2-methylphenyl)benzenesulfonamide	-35.55
IRA04	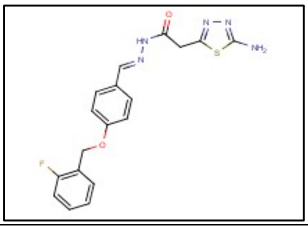	$C_{18} H_{16} F N_5 O_2 S$	2-(5-amino-1,3,4-thiadiazol-2-yl)-N'-{4-[(2-fluorobenzyl)oxy]benzylidene}acetohydrazide	-40.14
IRA05	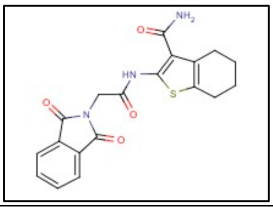	$C_{19} H_{17} N_3 O_4 S$	2-{[(1,3-dioxo-1,3-dihydro-2H-isoindol-2-yl)acetyl]amino}-4,5,6,7-tetrahydro-1-benzothiophene-3-carboxamide	-32.08

IRA06	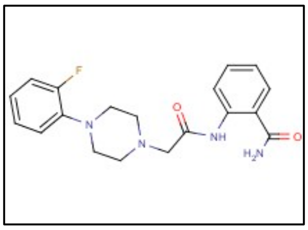	$C_{19} H_{21} F N_4 O_2$	2-({[4-(2-fluorophenyl)-1-piperazinyl]acetyl}amino)benzamide	-31.81
IRA07	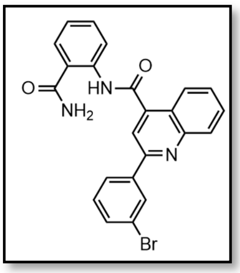	$C_{23} H_{16} Br N_3 O_2$	N-[2-(aminocarbonyl)phenyl]-2-(3-bromophenyl)-4-quinolinecarboxamide	-37.00
IRA08	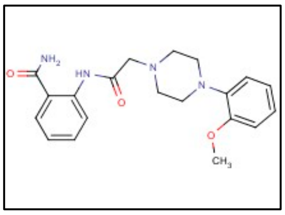	$C_{20} H_{24} N_4 O_3$	2-({[4-(2-methoxyphenyl)-1-piperazinyl]acetyl}amino)benzamide	-38.09
IRA09	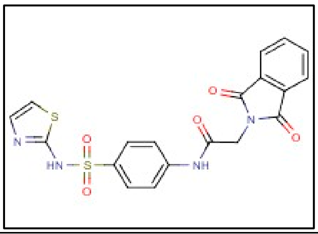	$C_{19} H_{14} N_4 O_5 S_2$	2-(1,3-dioxo-1,3-dihydro-2H-isoindol-2-yl)-N-{4-[(1,3-thiazol-2-ylamino)sulfonyl]phenyl}acetamide	-38.21
IRA10	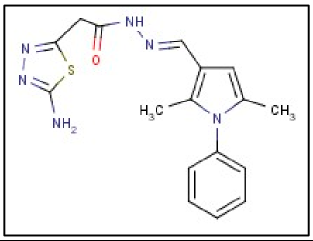	$C_{17} H_{18} N_6 O S$	2-(5-amino-1,3,4-thiadiazol-2-yl)-N'-[(2,5-dimethyl-1-phenyl-1H-pyrrol-3-yl)methylene]acetohydrazide	-37.84

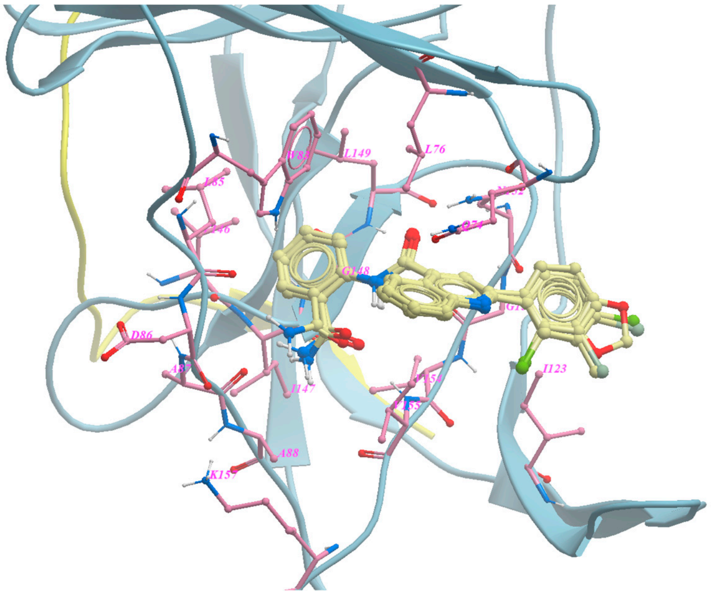

Figure S1. Predicted binding conformations of RI07, RI22, RI23, RI24, RI27, and RI28 targeting the open conformation of NS2B-NS3 protease at the identified allosteric pocket.

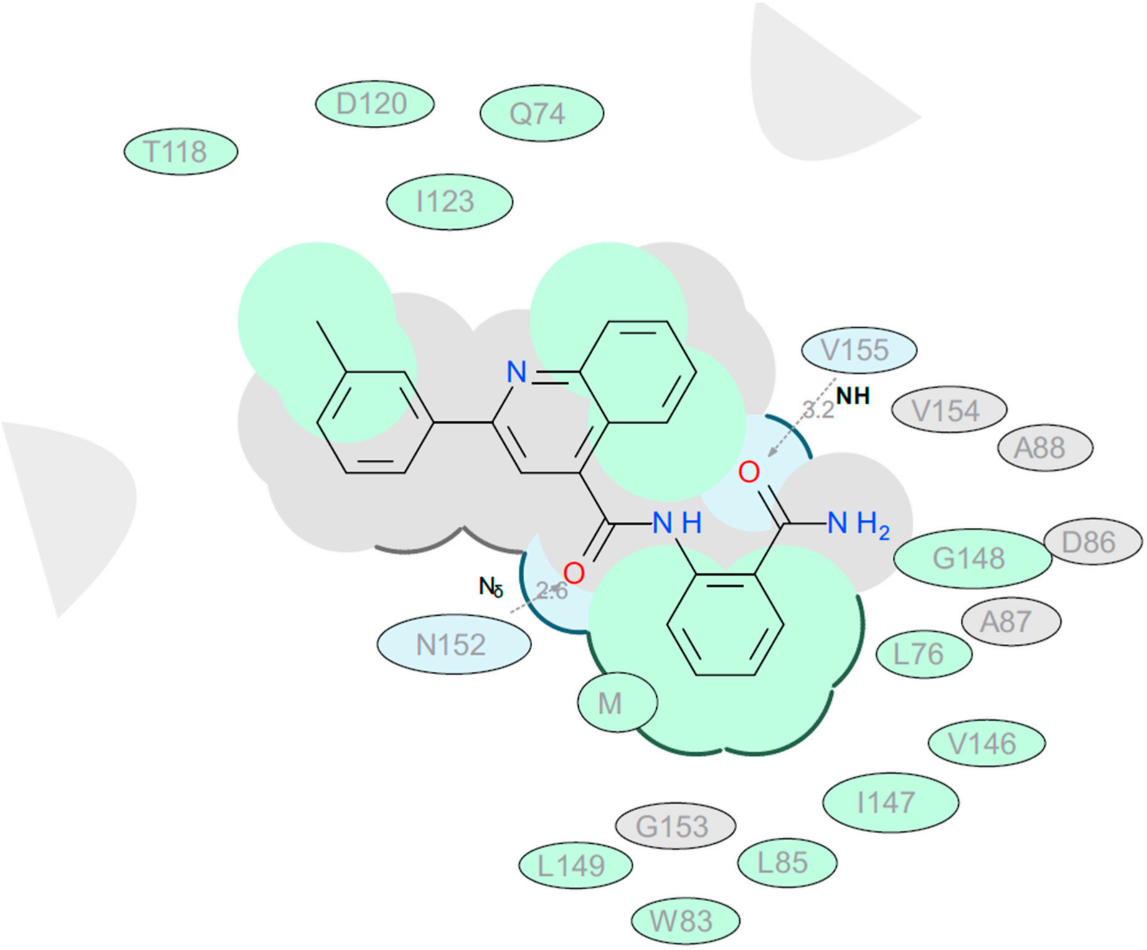

Figure S2. 2D interaction diagram for predicted pose of RI22 compound (L76, W83, L85, D86, A87, A88, T118, D120, I123, V146, I147, G148, L149, N152, G153, V154, V155). Hydrogen bonding interactions are shown by grey dotted lines.

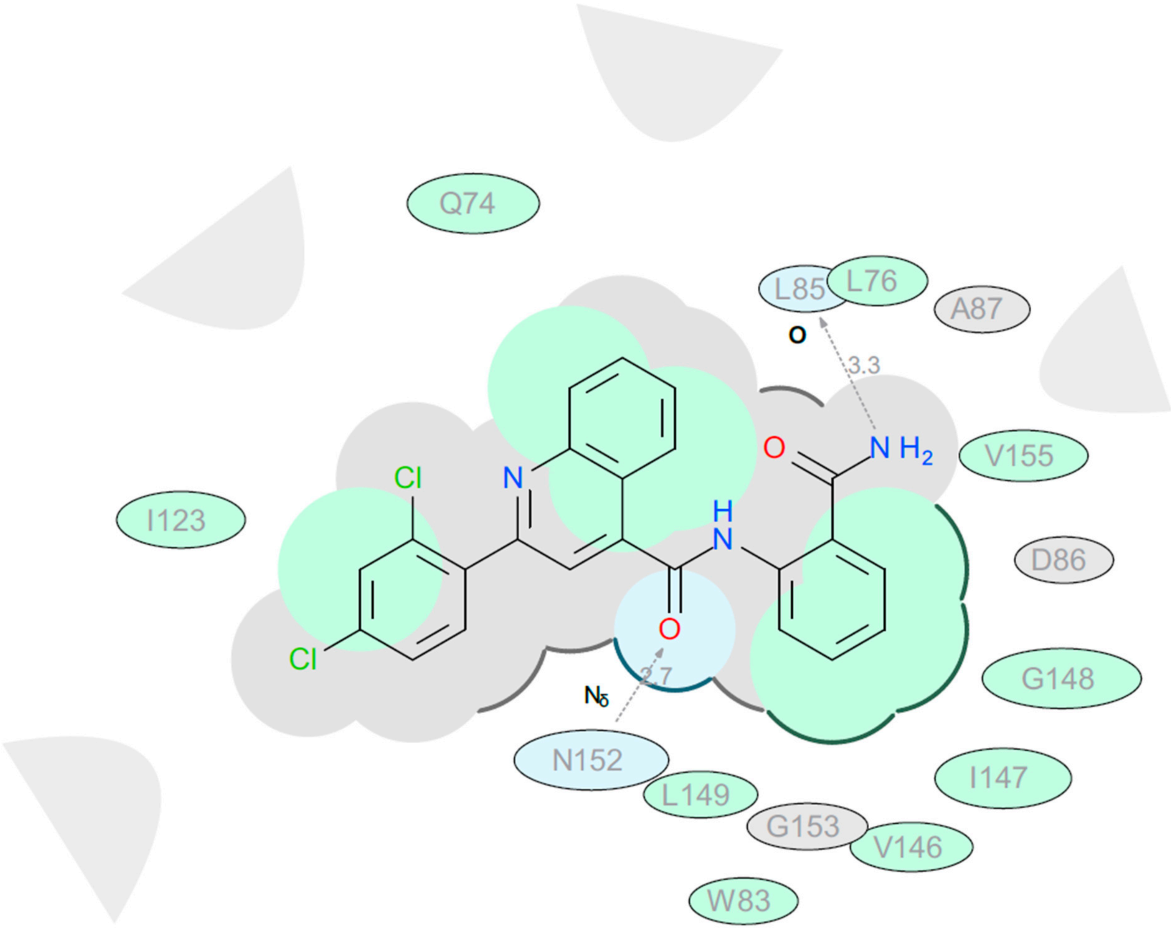

Figure S3. 2D interaction diagram for predicted pose of RI23 compound (Q74, L76, W83, L85, D86, A87, I123, V146, I147, G148, L149, N152, G153, V155). Hydrogen bonding interactions are shown by grey dotted lines.

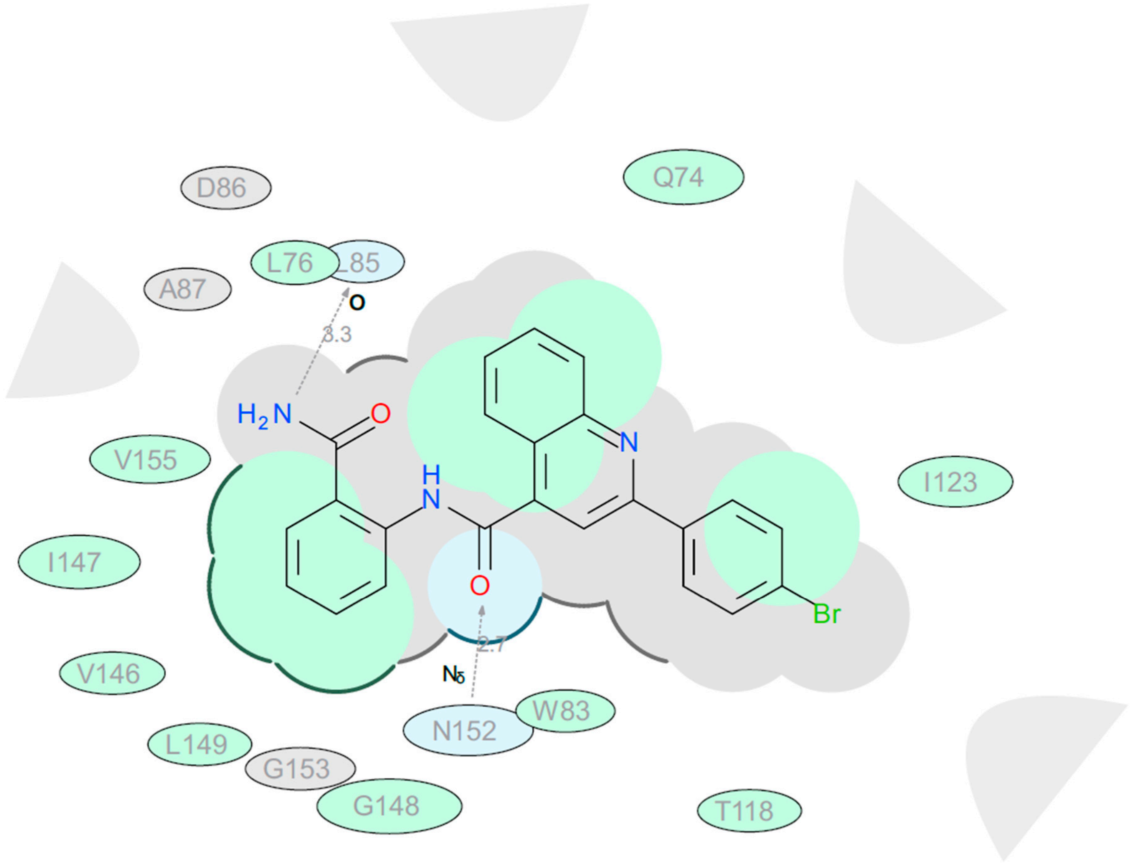

Figure S4. 2D interaction diagram for predicted pose of RI24 compound (Q74, L76, W83, L85, D86, A87, T118, I123, V146, I147, G148, L149, N152, G153, V155). Hydrogen bonding interactions are shown by grey dotted lines.

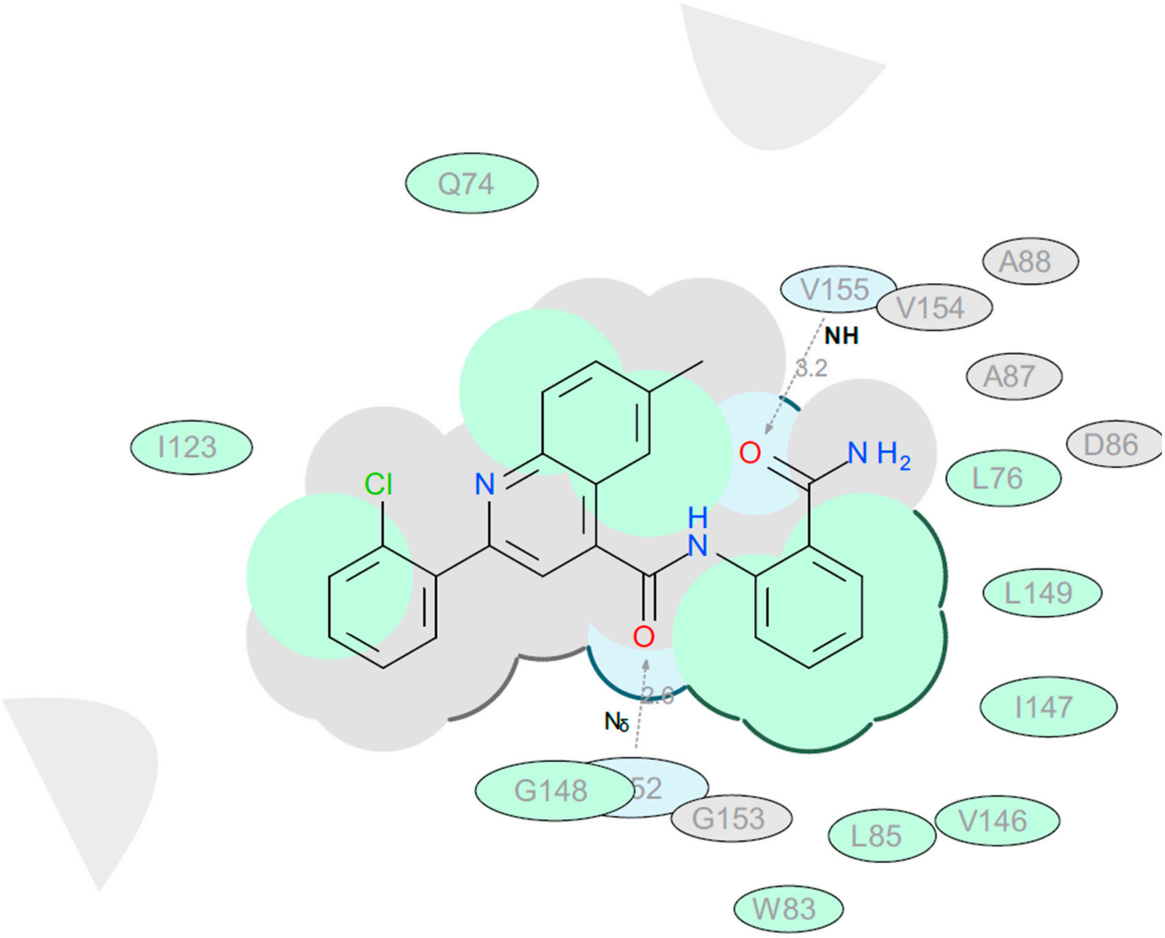

Figure S5. 2D interaction diagram for predicted pose of RI27 compound (Q74, L76, W83, L85, D86, A87, A88, I123, V146, I147, G148, L149, N152, G153, V154, V155). Hydrogen bonding interactions are shown by grey dotted lines.

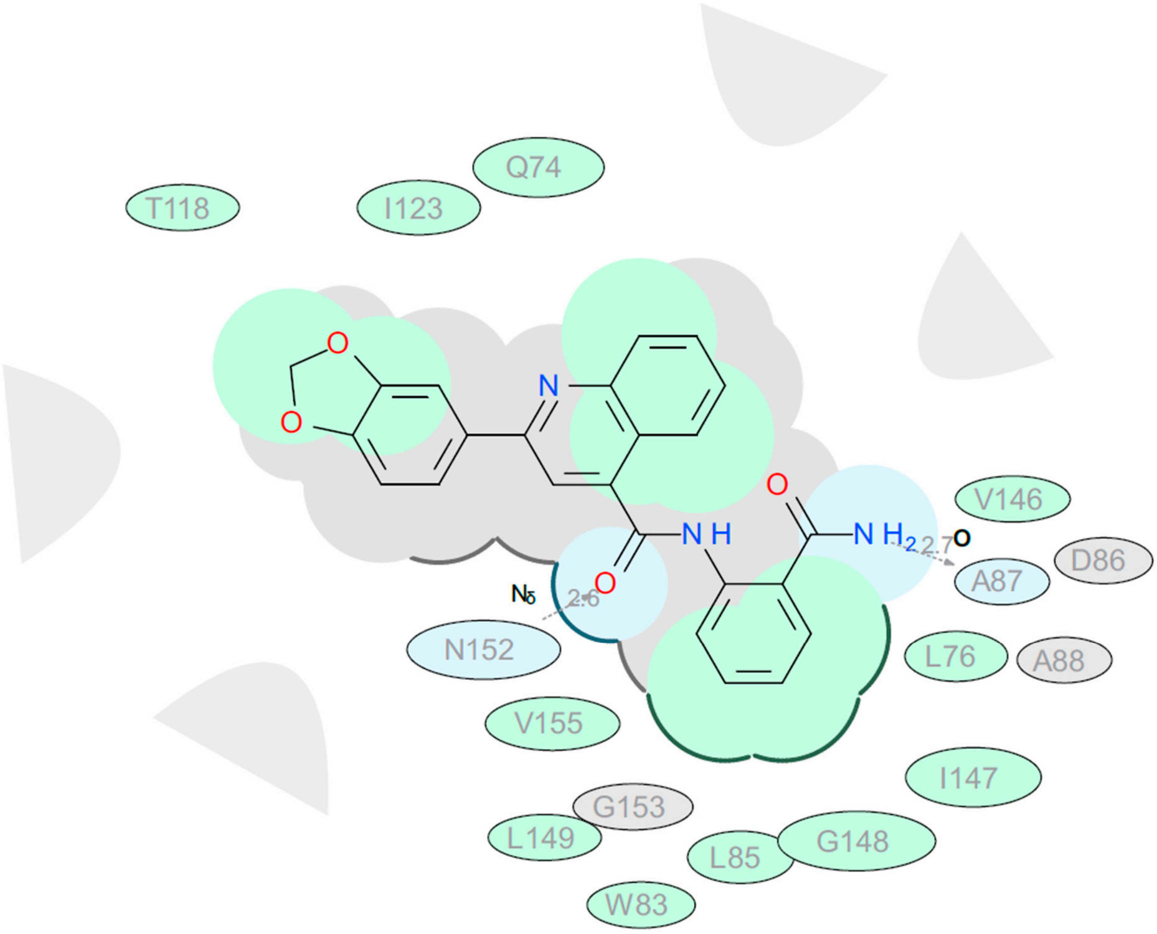

Figure S6. 2D interaction diagram for predicted pose of RI28 compound (Q74, L76, W83, L85, D86, A87, A88, T118, I123, V146, I147, G148, L149, N152, G153, V154, V155). Hydrogen bonding interactions are shown by grey dotted lines.
